# Efficacy Trial of a Mobile Application for Fluid Intake Management in Patients Receiving Chronic Hemodialysis Therapy

**DOI:** 10.1016/j.xkme.2026.101443

**Published:** 2026-06-17

**Authors:** Michael Rocco, Marion Rigaud, Carole Ertel, Greg Russell, Juliane Zemdegs, Mariacristina Vecchio

**Affiliations:** 1 Section on Nephrology, Wake Forest School of Medicine, Winston-Salem, NC; 2 Danone Research & Innovation, Gif-sur-Yvette, France; 3 Wake Forest Outpatient Dialysis, High Point, NC; 4 Department of Biostatistics and Data Science, Wake Forest School of Medicine, Winston-Salem, NC

**Keywords:** Behavioral control techniques, fluid intake, hemodialysis, interdialytic weight gains, mobile application

## Abstract

**Rationale & Objective:**

Increased interdialytic weight gain (IDWG) is associated with increased morbidity and mortality in patients receiving chronic hemodialysis. We evaluated the efficacy of a smartphone-based application (app) designed to support reductions in interdialytic fluid intake.

**Study Design:**

Pre–post intervention study.

**Setting & Participants:**

Adult patients receiving chronic hemodialysis with mean IDWG >3.5% over a 3-month period were recruited from outpatient hemodialysis units in central North Carolina, United States.

**Intervention:**

Participants were trained to use a smartphone-based app that allowed recording fluid intake and incorporated behavioral support features (eg, goal setting, prompts, feedback). The patients were asked to use the app daily during the 8-week active phase of the trial.

**Outcomes:**

The primary study outcome was the change in mean IDWG from baseline (before app usage) to the 8-week active phase. Secondary outcomes included app safety and usage. IDWG was also assessed during a 6-month passive phase to monitor for possible long-term behavioral changes.

**Results:**

Twenty-three patients completed the 8-week active phase and provided app data. Compared with baseline, mean IDWG for the 3-day interdialytic interval decreased by −0.52 ± 0.24 lb (*P* = 0.032) during the active phase and −0.86 ± 0.96 lb (*P* < 0.01) during the passive phase. No safety concerns (defined as >50% increase in IDWG) were observed. During the active phase, app was used on ≥80% of the days by 65% of participants and daily by 26% of participants. Reductions in IDWG were sustained during the passive phase. Correlations between app-recorded fluid intake and IDWG were weak and not statistically significant.

**Limitations:**

This uncontrolled pre–post study was subject to selection bias toward more technologically literate and adherent participants, did not meet recruitment targets, and lacked urine output data.

**Conclusions:**

Use of a smartphone-based app designed to support fluid intake restriction is feasible and associated with sustained reductions in IDWG among patients receiving chronic hemodialysis, without safety concerns.

## Plain-Language Summary

Excessive weight gains between hemodialysis sessions increase the risk of hospitalization and death. We developed a smartphone app that allows patients to record their fluid intake on a daily basis. The app also provides practical tips to help limit fluid intake and grants digital rewards when goals are met. We found that, on average, patients gained less weight between dialysis sessions while using the app compared with the period before they used it. These results suggest that smartphones apps that combine fluid intake tracking with behavioral support may help patients better control their fluid intake between dialysis sessions.

Large observational studies across multiple countries have consistently shown that high interdialytic weight gain (IDWG) is associated with increased morbidity and mortality in patients receiving chronic hemodialysis, both after adjustment for body weight and independent of body weight.^1^, ^2^, ^3^, ^4^, ^5^ Despite the clinical importance of fluid restriction, nonadherence remains common, with prevalence estimates ranging from 30% to 60% among hemodialysis patients.^6^, ^7^, ^8^ Contributing factors include poor adherence to fluid restrictions, high sodium intake, thirst, and xerostomia.^6^

A variety of behavioral techniques have been attempted to limit IDWG in this population^9^; however, their effectiveness has been inconsistent, and sustained adherence remains challenging. Importantly, although several smartphone applications (apps) are available to track fluid intake, we are not aware of any that integrate evidence-based behavioral techniques alongside intake monitoring to actively support adherence to fluid restriction in patients receiving hemodialysis.

Although numerous mobile apps are available to encourage increased water intake, there is a paucity of apps designed to help individuals limit the fluid intake due to medical necessity. A literature review published in 2019 identified 15 apps designed to assist patients in the self-management of their chronic kidney disease.^10^ Of these, only 2 included functionalities to evaluate fluid intake, and both were limited to passive recording than active behavioral support (D-Track: Dialysis Tracker [now called Dialysis Pro]^11^ and H2O Overload^12^). Similarly, a separate 2019 review of kidney disease-related mobile apps available since 2014 identified 28 patient-facing apps across Android and iOS platforms, of which only 6 targeted patients with end-stage kidney disease, and none were specifically designed to manage fluid intake.^13^ In addition, several apps identified in earlier studies are either no longer available or have not achieved widespread distribution.^14^, ^15^, ^16^

In the present study, we developed a smartphone-based app specifically designed to support fluid intake restriction in patients receiving chronic hemodialysis. The app incorporates several behavioral change techniques intended to promote reductions in fluid intake, even when used intermittently.^17^ Its safety, usability, and design were tested in a feasibility study, which informed iterative refinements to both behavioral components and user data entry process.^18^ Here, we report the results of the subsequent efficacy trial (ClinicalTrials.gov identifier NCT03759847) assessing whether use of the app was associated with a reduction in the mean IDWG in patients undergoing chronic hemodialysis.

## Methods

### Participants

Adults (aged ≥18 years) with end-stage kidney disease undergoing chronic hemodialysis were recruited from Wake Forest outpatient hemodialysis units (North Carolina, USA). The study was approved by the Wake Forest Institutional Review Board, and all participants provided written informed consent before any study procedures.

Inclusion criteria were a 30-day average IDWG ≥3.5% of body weight. This cut point was based on the National Kidney Foundation Kidney Disease Outcomes Quality Initiative (KDOQI) guidelines for hemodialysis adequacy in 2006 indicating IDWG should remain less than 4.0% to 4.5% of dry weight.^19^ Participants were also required to have access to an iOS or Android smartphone and to be comfortable with using apps on a regular basis. Exclusion criteria included planned living-donor kidney transplantation within 4 months, heart failure class III or IV as per New York Heart Association classification,^20^ need for chronic oxygen therapy due to pulmonary disease, or hospitalization within 30 days before study enrollment.

Participants were trained by the study coordinator to use the app, including reviewing app features and setting an individualized daily fluid intake goal, defined in consultation with their primary nephrologist, based on clinical parameters such as body weight and blood pressure.

### Mobile App Development

The app was developed for iOS and Android smartphones and designed to support fluid intake restriction using several behavioral change techniques derived from the Behavioral Change Technique Taxonomy as ingredients to elicit change.^17^ The behavioral change techniques integrated into the app were selected for their relevance to address study objectives and included goal setting, action planning, self-monitoring, feedback on behavior and outcomes, prompts/cues, information on health consequences, and social rewards earned for consistency of tracking and accuracy of behavior. Details of the app’s development and embedded behavioral change techniques have been reported previously.^18^

### Study Visits and Data Collection

**Figure 1:**
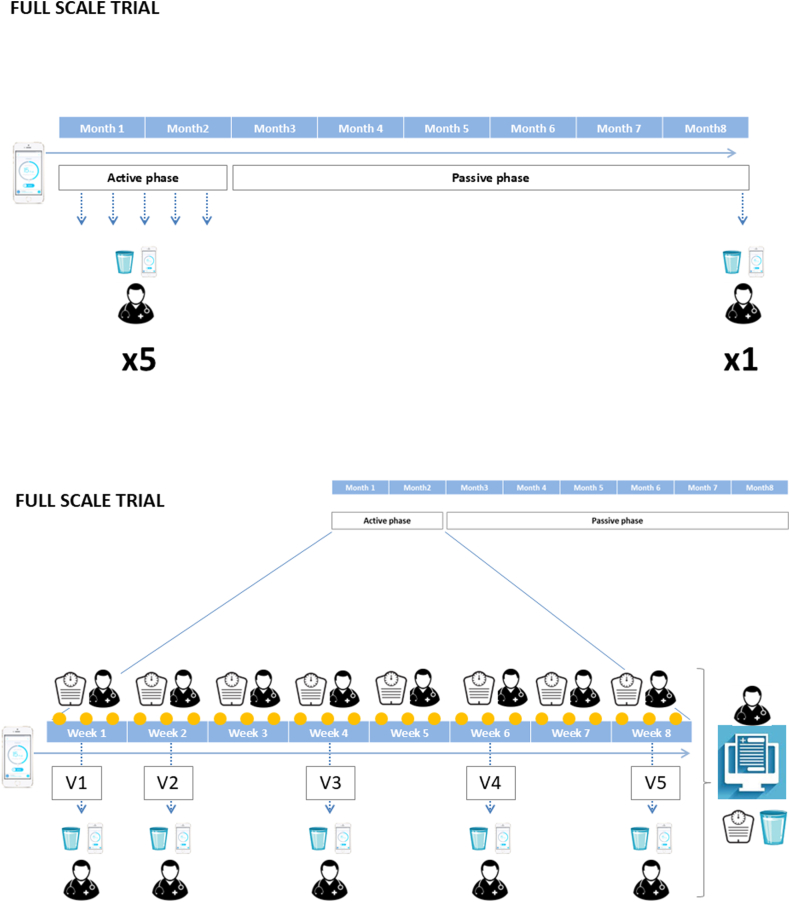
Study timeline. Over a 1-month period, participants recorded their daily fluid intake by means of the mobile app. Dialysis sessions happened every 2 days during weekdays. Participants’ body weight was measured and recorded before and after every dialysis session. Study visits were conducted at weeks 1 and 4. Yellow dot indicates a dialysis session. Scale icon indicates that pre- and post-dialysis body weight were measured and recorded. Person with stethoscope icon indicates that a survey was conducted by the study coordinator with the participant. Glass icon indicates review of FiApp data by the study coordinator.

The study consisted of an 8-week active phase, during which participants were encouraged to use the app daily, followed by a 6-month passive phase with optional app use (Fig 1). During the active phase, participants were asked to record their daily fluid intake in the app. Fluid intake data could be exported by participants and emailed to the study coordinator; data was not stored on a central server.

Hemodialysis sessions occurred 3 times per week (two 2-day and one 3-day interdialytic intervals). Participants’ body weight was measured before and after each dialysis session using a calibrated floor scale in the dialysis unit, and IDWG was calculated accordingly. Study visits were conducted at weeks 1, 2, 4, 6, and 8 of the active phase to ascertain app use and answer any participant questions. Surveys assessing app usability and perceptions were completed at each active-phase visit and at the end of the passive phase, using a 5-point Likert scale.

### Primary and Secondary Outcomes

The primary outcome was change in mean IDWG from baseline (pre-app) to both the active (months 1-2) and the passive (months 3-8) phases of app use. Secondary outcomes included app safety, utilization, and the correlation between app-recorded fluid intake and IDWG. Safety was defined as the absence of a ≥50% increase in IDWG during use of the app compared to baseline. Utilization was assessed as the number and proportion of days used and by day-of-week patterns, with the expectation of similar usage across days.

### Sample Size

The study was powered to detect a 20% decrease in mean IDWG from the pre-app period to the period of app use. For a 2-day interdialytic interval, a sample size of 42 participants would have 80% power to detect a 20% difference in means of 0.94 (pre-app weight removal of 4.7 lb and a post-app removal of 3.76 lb) during the active phase, assuming a standard deviation of differences of 2.11, using a paired *t* test with a 0.05 two-sided significance level. For a 3-day interdialytic interval, a sample size of 42 would have 80% power to detect a 16% difference in means of 0.94 (pre-app weight removal of 5.7 lb and a post-app removal of 4.56 lb) during the active phase, assuming a standard deviation of differences of 2.11, using a paired *t* test with a 0.05 two-sided significance level. To account for dropouts, a target enrollment of 55 participants was set.

### Statistical Analysis

Descriptive statistics were used to summarize participant’s demographic and baseline clinical characteristics. Normality of continuous variables was assessed using the Shapiro-Wilk W test, and homoscedasticity was evaluated using a likelihood ratio test.

IDWG was calculated for each participant across the 3 study periods (pre-app, active phase, and passive phase). Differences in estimated (least squares) mean IDWG across study periods were calculated using repeated measures regression. Specifically, a repeated measures analysis of variance with an unstructured covariance matrix was used to account for within-participant correlation over time when comparing mean IDWG across study periods.

The association between app-recorded fluid intake and IDWG during the active phase was evaluated by calculating the ratio of app-recorded fluid intake to IDWG between 2 dialysis sessions, and correlation was assessed using Pearson correlation coefficient. This analysis was also performed in the high user’s subset, defined as participants who used the app on at least 80% of the study days during the active phase.

To evaluate app safety, the average 3-month IDWG pre-app use was compared with the average 8-week IDWG during the active phase. In addition, the percentage change in body weight during app use was calculated.

App usage patterns across the 7 days of the week were compared, and Fisher exact test was used to assess differences in the proportion of readings observed. Repeated measures models were used to app usage data to assess longitudinal changes in survey responses.

All statistical analyses were performed at the same alpha level of 0.05. Adjustment for multiple comparisons was performed using the Tukey test where applicable. All analyses were performed using SAS version 9.4.

## Results

### Participant Flow and Baseline Characteristics

Thirty-eight individuals consented and 23 completed the active phase of the trial. Of the 15 patients who did not complete the active trial, 6 withdrew without providing data, 2 changed to peritoneal dialysis, 4 withdrew after starting the study due to personal issues, and 3 withdrew due to issues related to their smartphones. Five participants did not complete the passive phase of the trial: 1 changed to peritoneal dialysis, 1 due to kidney transplantation, and 3 for personal reasons. The participant’s demographic and baseline clinical characteristics are described in Table 1. Median age was 56 ± 13.6 years, 43% were female, 61% self-identified as Black, and the median chronic hemodialysis duration was 2.2 years (range, 0.3-20.2 years).

**Table 1:** Demographic and Baseline Clinical Characteristics of Participants Who Completed the 8-Week Active Study Period

Baseline Characteristics	n (%)
**Sex**	
Men	13 (57%)
Women	10 (43%)
**Duration of chronic hemodialysis (mo)**	
<12	5 (22%)
12-48	12 (52%)
>48	6 (26%)
**Body mass index (kg/m^2^)**	
Underweight/normal (<25)	5 (22%)
Overweight (25.1-29.9)	6 (26%)
Obese (30+)	12 (52%)
**Comorbid conditions**	
Glomerulonephritis	2 (9%)
Hypertension	5 (22%)
Type 2 diabetes mellitus	12 (52%)
Other	4 (17%)

### Interdialytic Weight Gain

Mean IDWG declined over the trial (Table 2). For the 2-day intervals, IDWG decreased from 6.10 lb before the study to 5.90 lb during the active phase and 5.47 lb during the passive phase. For the 3-day intervals, IDWG decreased from 8.96 lb before the study to 8.41 lb (active phase) and 8.12 lb (passive phase). Repeated measures analysis showed a statistically significant decline over time for both intervals (*P* < 0.01 for each). Monthly and participant-level data are provided in Tables S1, S2a and b.

**Table 2:** Interdialytic Weight Gain (IDWG; in Pounds) by Study Period

Days Between	N Obs	Variable	N	Mean	SD	25th Pctl	Median	75th Pctl
2	22	Mean pre Mean app use Mean passive	22 22 22	6.10 5.90 5.47	1.55 2.08 2.04	5.00 4.61 4.41	5.85 5.71 5.21	6.76 6.64 6.14
3	23	Mean pre Mean app use Mean passive	23 23 23	8.96 8.41 8.12	1.75 2.10 2.22	7.83 6.79 6.53	8.38 7.87 8.00	9.91 9.91 9.99

*Note:* The estimates below were calculated by the repeated measures regression models.

**Overall *P* value for 2-day intervals: *P* = 0.0001.**

**2-day intervals (LS estimated mean ± SE, 95% CI):**

Pre: 6.06 ± 0.37 (95% CI, 5.32-6.81).

App use: 5.88 ± 0.37 (95% CI, 5.12-6.63).

Passive: 5.48 ± 0.37 (95% CI, 4.74-6.23).

**2-day intervals – Estimated differences:**

Passive − Active: −0.39 ± 0.14 (95% CI, −0.66 to −0.12), *P* = 0.0061.

Active − Pre: −0.19 ± 0.14 (95% CI, −0.46 to 0.09), *P* = 0.19.

Passive − Pre: −0.58 ± 0.12 (95% CI, −0.83 to −0.33), *P* < 0.0001.

**Overall *P* value for 3-day intervals: *P* = 0.0009.**

**3-day intervals (LS estimated mean ± SE, 95% CI):**

Pre: 8.96 ± 0.41 (95% CI, 8.13-9.79).

App use: 8.44 ± 0.41 (95% CI, 7.58-9.29).

Passive: 8.11 ± 0.41 (95% CI, 7.28-8.93).

**3-day intervals – Estimated differences:**

Passive − Active: −0.33 ± 0.23 (−0.81 to 0.14), *P* = 0.16.

Active − Pre: −0.52 ± 0.24 (−1.00 to −0.05), *P* = 0.032.

Passive − Pre: −0.86 ± 0.21 (−1.28 to −0.43), *P* = 0.0002.

Abbreviations: CI, confidence interval; LS, least squares; Obs, observations; Pctl, percentile; SD, standard deviation; SE, standard error.

### Correlation of IDWG with Recorded Fluid Intake

**Figure 2:**
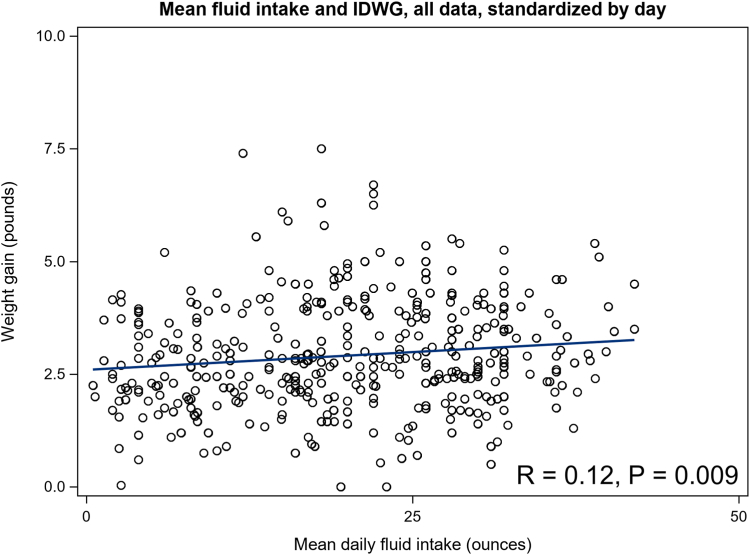
Relationship between mean interdialytic weight gain (IDWG) and mean app-recorded daily fluid intake in ounces for all participants.

**Figure 3:**
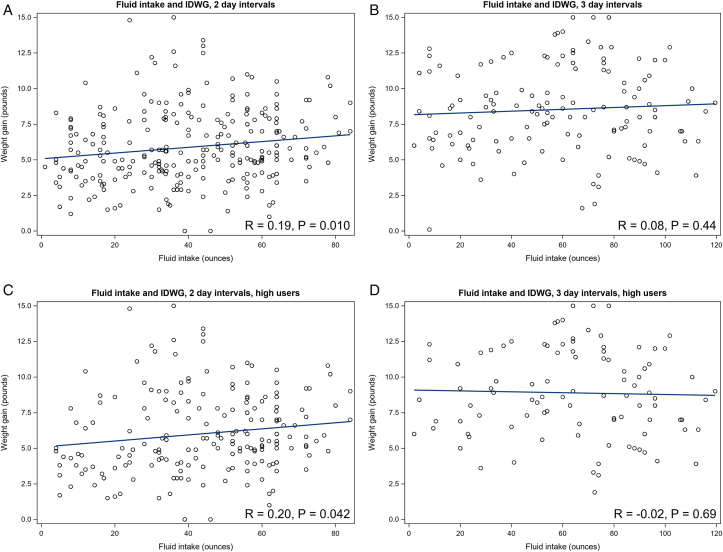
Relationship between mean interdialytic weight gain (IDWG) and mean app-recorded daily fluid intake in ounces by subgroups: (A) Two-day interdialytic period. (B) Three-day interdialytic period. (C) Two-day interdialytic period for high users of the FiApp. (D) Three-day interdialytic period for high users of the FiApp. High users of the FiApp were defined as participants who used the FiApp on at least 80% of the study days during the active phase.

Despite reduced IDWG, correlations between app-recorded fluid intake during the active phase and IDWG were weak (*R* = 0.12; *P* < 0.01) (Fig 2). A weak correlation was observed for 2-day intervals (*R* = 0.19, *P* = 0.01), with no correlation for 3-day intervals (*R* = 0.08, *P* = 0.44; Fig 3a and b). Analysis of high users’ subset also showed no correlation. App-recorded fluid intake to IDWG ratios varied widely, ranging from 0.07 to 0.96 (Table 3), consistent with variable use of the app to record fluid intake, as noted below.

**Table 3:** Ratio of App-Recorded Fluid Intake to IDWG by Dialytic Interval

Dialytic Interval	Ratio of App-Recorded Fluid Intake to IDWG				
	Mean	Standard Deviation	Median	Minimum	Maximum
2-d interdialytic interval	0.26	0.24	0.18	0.07	0.96
3-d interdialytic interval	0.30	0.28	0.21	0.05	1.15
Overall data	0.27	0.22	0.24	0.07	0.96

Abbreviation: IDWG, interdialytic weight gain.

### App Usage During the Active Phase

During the active phase, the app was used on ≥80% of the days by 65% of participants and daily by 26%. The median number of days the app was used was 89.2%. On average, participants were enrolled in the active phase of the study for 52.9 ± 6.2 days and recorded fluid intake data on 42.3 ± 13.7 days. The median number of days with recorded fluid intake was 89.3% (interquartile range, 63%-100%). App use was similar across weekdays and weekends (Table S3). The lowest rate of use was observed on Wednesday (77.5%) compared with a high-water mark of 82.6% on Thursday and Saturday; day-to-day differences were not significant (*P* = 0.28).

Despite the app being used at a consistently high daily rate, data recorded per day was inconsistent, with minimum recorded intake of ≤8 fl oz in 11 of 23 participants (2-day interval) and 9 of 23 participants (3-day interval). Table 4 depicts the app-recorded fluid intake in ounces by participant for 2-day and 3-day interdialytic intervals.

**Table 4:** App-Recorded Fluid Intake in Ounces by Participant for 2-Day and 3-Day Interdialytic Interval

Dialytic Interval	Mean	Standard Deviation	Minimum	Maximum
2-d interdialytic interval	40.1	17.9	3.1	82.5
3-d interdialytic interval	55.2	31.2	11.6	124.7

Although not statistically significant (*P* = 0.08), participant survey data showed a decrease in app use over time, with the proportion of participants reporting using the app ≥1 time daily declining from 85% at week 1 to 62% at month 8. After adjusting for multiple comparisons (Tukey method), no pairwise differences from baseline were statistically significant (Table 5).

**Table 5:** Frequency of App Use as Reported in Participant Surveys

Frequency	Week 1	Week 2	Week 4	Week 6	Week 8	Month 8	Total
More than once/day	22 (85%)	20 (74%)	20 (77%)	16 (76%)	14 (64%)	13 (62%)	105
Once/day	1 (4%)	3 (11%)	0 (0%)	0 (0%)	2 (9%)	2 (10%)	8
3+ times/week	3 (12%)	3 (11%)	4 (15%)	2 (10%)	4 (18%)	2 (10%)	18
Once a week	0 (0%)	1 (4%)	2 (8%)	2 (10%)	0 (0%)	0 (0%)	5
Once/month	0 (0%)	0 (0%)	0 (0%)	0 (0%)	0 (0%)	1 (5%)	1
<1 month	0 (0%)	0 (0%)	0 (0%)	1 (5%)	2 (9%)	3 (14%)	6
Total	26	27	26	21	22	21	143

*Note:* Adjusted *P* values (Tukey method) for comparisons with week 1 were as follows: week 2, *P* = 0.65; week 4, *P* = 0.76; week 6, *P* = 0.70; week 8, *P* = 0.55; month 8, *P* = 0.18.

### App Safety

Individual participant data on mean IDWG for the 2-day and 3-day intervals are depicted in Table S2a and b, respectively. One participant experienced a ≥20% transient IDWG increase during active app use, which resolved during the passive phase.

### User Feedback

Survey completion exceeded 95%. Over 75% of the participants rated app functions positively, with stable ratings over time for ≥90% of the survey categories (data not shown). Of the participants, 27.7% agreed and 63.5% totally agreed they would recommend the app (Table 6). The survey instrument is reproduced in Table S4.

**Table 6:** Likert Scale Ratings of the Fluid Intake App Based on Participant Survey Responses

	Percentage of Participants					*Number of Responses*
	Totally Disagree	Disagree	Neutral	Agree	Totally Agree	
The application is easy to use	0	0	6.6	37.8	55.6	151
I learned quickly how to use the app	0	3.3	4.7	38.7	53.3	150
The app helps me monitor accurately how much I drink	0	0.7	4.7	38.7	56.0	150
The app helps me understand why I have to reduce my fluid consumption	0	1.3	8.0	37.1	53.6	151
The app increases my motivation to meet my fluid intake target	0	0	6.6	38.4	55	151
The app helps me regulate my fluid consumption	0	0	6.0	40.7	53.3	150
The app helps me reach my fluid intake target	0	0	15.3	34.7	50.0	150
I would recommend the app to another patient	0	2.7	6.1	27.7	63.5	148
***The following parts of the app are easy to use:***						
Fluid intake tracker	0	0	4.0	39.6	56.4	149
Strategy planning	0	0	16.8	39.9	43.4	143
Personal statistics	0	0	12.8	39.6	47.7	149
Export of statistics	0	1.4	17.0	38.1	43.5	147
Health related information (tips)	1.3	10.1	40.3	44.3	4.0	143
Application badges (rewards)	1.6	0	16.4	37.5	44.5	128
***It is easy to learn how to use the following parts of the app:***						
Fluid intake tracker	0.7	0	2.6	42.4	54.3	151
Strategy planning	0.7	0	11.6	41.8	45.9	146
Personal statistics	0.7	0	11.9	40.4	47.0	151
Export of statistics	0.7	0	13.0	41.1	45.2	146
Health related information (tips)	1.4	0	10.1	34.5	54.0	139
Application badges (rewards)	1.6	0	11.3	33.9	53.2	124
***The following parts of the app are a motivation to regulate my fluid consumption:***						
Fluid intake tracker	0	0	5.3	46.4	48.3	151
Strategy planning	0	0.7	17.1	42.5	39.7	146
Personal statistics	0	0	18.1	43.6	38.3	149
Export of statistics	1.4	0.7	18.6	42.8	36.6	145
Health related information (tips)	1.4	0.7	15.9	43.4	38.6	145
Application badges (rewards)	1.6	1.6	21.1	41.4	34.4	128
***In my opinion, the following parts of the app are essential to the app:***						
Fluid intake tracker	0	0	2.0	36.4	61.6	151
Strategy planning	0	0.7	11.5	44.6	43.2	148
Personal statistics	0	0	8.7	44.7	46.7	150
Export of statistics	0.7	1.4	12.2	41.2	44.6	148
Health related information (tips)	0	0.7	12.8	43.9	42.6	148
Application badges (rewards)	0	4.6	19.2	38.5	37.7	130

## Discussion

We demonstrate that using our fluid intake app was associated with a statistically significantly decreased IDWG among chronic hemodialysis patients, a result we also believe to be clinically meaningful. We attribute these results not only to the user interface, which was improved based on participants’ feedback during the feasibility study, but also possibly to the integration of behavioral change techniques, including goal setting, action planning, self-monitoring, tailored feedback on behaviors and outcomes, prompts addressing thirst and xerostomia, and social reinforcement through reward badges.^21^ Survey data support this interpretation, as participants reported that the app improved their understanding of why fluid intake restriction is important, increased their motivation to meet fluid intake targets, and helped them reach the assigned fluid goals. Together, these findings suggest that the behavioral components of the app played an important role in helping decrease IDWG beyond fluid intake monitoring.

Sustained engagement with the app further supports the relevance of these behavioral techniques. Three-quarters of participants reported using the app more than once daily during the active phase, and over 60% continued this level of engagement at 8 months. This pattern suggests that behavioral prompts, feedback, and rewards remained relevant throughout the study period. Importantly, this sustained engagement occurred despite variability in the completeness of fluid intake recording, indicating that the app’s impact on behavior may extend beyond accurately logging fluid intake per se.

Traditional behavioral approaches to reduce IDWG, such as frequent staff telephone contact, group education sessions, and cognitive behavioral therapy, are often resource-intensive, limiting their scalability in routine dialysis units. In contrast, the fluid intake app requires minimal staff involvement, can be deployed rapidly, typically requiring ≤5 minutes of patient training, and it enables continuous, low-burden behavioral support integrated into daily life. By embedding evidence-based behavioral techniques that specifically address fluid restriction challenges in dialysis patients, this app offers a potential scalable alternative to labor-intensive interventions.

Multiple reviews demonstrate the limited availability and functionality of mobile apps for patients with chronic kidney disease. A US review of Apple and Google platforms (June-July 2020) identified 10 nutrition-related chronic kidney disease apps, only 2 of which were available on both platforms, and none reported the ability to track fluid intake.^21^ An earlier US survey (2016-2017) identified 12 apps supporting nutrition or medication tracking; only 2 (H2O Overload and D-Track: Dialysis Tracker) monitored fluid intake, and none incorporated advanced support features such as motivational feedback or responses to measured water, weight, or phosphorus levels.^22^

Similar limitations were observed internationally. An Australian review^23^ identified 21 kidney nutrition apps, over half of which were inaccurate or not evidence-based; although some provided self-monitoring guidance, none enabled in-app fluid tracking. A 2018 review^24^ of dialysis diet apps in the Asian market identified 22 apps, fewer than half meeting inclusion criteria, with limited functionality and no detailed reporting on fluid control features. Finally, a literature and gray literature review identified only 9 apps supporting dietary self-monitoring for individuals with chronic kidney disease; among the 3 addressing weight gain or fluid intake, none were published after 2013, and most were developed for outdated platforms.^14^, ^15^, ^16^^,^^25^ These findings informed the design of our app, which integrates behavioral support elements such as reminders, personalized notifications, and incentives to promote adherence beyond fluid intake tracking.

Limitations of the study warrant consideration. First, the pre–post study design without a concurrent control group raises the possibility of a Hawthorne effect, whereby participants modify behavior simply due to awareness of being observed. Although such an effect cannot be excluded, the persistence of reduced IDWG during the 6-month passive phase, when app use was optional and study contact was reduced, argues against a purely short-term observation effect. This sustained reduction suggests that longer-lasting behavioral changes may have been established during the active phase. Second, the study design required participants to have access to and be comfortable using a smartphone, which may have introduced selection bias favoring individuals who are more technologically literate, more engaged in self-management, or inherently more adherent. This bias may limit generalizability to patients with lower digital literacy or limited access to mobile technology. Additional limitations include failure to reach the planned sample size, lack of urine output data, incomplete reporting of fluid intake data, and poor correlation between fluid intake recorded in the app and IDWG. The latter likely reflects inconsistent logging rather than absence of behavioral change, underscoring the importance of behavioral cues and feedback independent of precise fluid intake tracking.

In conclusion, this study demonstrates the feasibility of using a smartphone app to support fluid intake restriction in chronic dialysis patients and suggests that integrating behavioral techniques into digital tools may contribute to clinically meaningful decrease in IDWG. Large randomized controlled trials are warranted to confirm efficacy, assess generalizability, and determine the relative contributions of fluid intake monitoring, behavioral support, and observation effects.
