# Supplementary material for: Efficacy Trial of a Mobile Application for Fluid Intake Management in Patients Receiving Chronic Hemodialysis Therapy
Source: Kidney Med. 2026 Jun 17;8(8):101443. doi: 10.1016/j.xkme.2026.101443 (PMC13396856; doi:10.1016/j.xkme.2026.101443)
Supplement: Supplementary File (PDF) — Table S1-S4. [file mmc1.pdf]

**Supplementary Material**

**Table S1. Monthly interdialytic weight gain (IDWG; pounds) stratified by interdialytic period.**

| Repeated measures estimates – 2-day interdialytic period | | | 95% Bound | |
|---|---|---|---|---|
| **Period** | **Est mean** | **SE** | **Lower** | **Upper** |
| Month 3 Pre | 5.93 | 0.37 | 5.19 | 6.67 |
| Month 2 Pre | 6.03 | 0.37 | 5.31 | 6.76 |
| Month 1 Pre | 6.22 | 0.37 | 5.50 | 6.94 |
| Days 1 to 28 | 5.78 | 0.36 | 5.07 | 6.50 |
| Days 29 to 56 | 6.00 | 0.37 | 5.27 | 6.73 |
| Month 1 Passive | 5.73 | 0.37 | 5.01 | 6.45 |
| Month 2 Passive | 5.54 | 0.37 | 4.82 | 6.27 |
| Month 3 Passive | 5.18 | 0.37 | 4.45 | 5.91 |
| Month 4 Passive | 5.25 | 0.37 | 4.52 | 5.98 |
| Month 5 Passive | 5.19 | 0.37 | 4.47 | 5.91 |
| Month 6 Passive | 5.39 | 0.39 | 4.63 | 6.15 |
| **Repeated measures estimates – 3 day interdialytic period** | | | **95% Bound** | |
| **Period** | **Est mean** | **SE** | **Lower** | **Upper** |
| Month 3 Pre | 9.08 | 0.48 | 8.14 | 10.03 |
| Month 2 Pre | 9.04 | 0.46 | 8.14 | 9.94 |
| Month 1 Pre | 8.70 | 0.46 | 7.80 | 9.61 |
| Days 1 to 28 | 8.24 | 0.45 | 7.35 | 9.13 |
| Days 29 to 56 | 8.67 | 0.47 | 7.74 | 9.60 |
| Month 1 Passive | 8.54 | 0.45 | 7.65 | 9.44 |
| Month 2 Passive | 8.25 | 0.47 | 7.33 | 9.17 |
| Month 3 Passive | 7.54 | 0.46 | 6.63 | 8.45 |
| Month 4 Passive | 7.44 | 0.47 | 6.52 | 8.36 |
| Month 5 Passive | 7.31 | 0.46 | 6.39 | 8.22 |
| Month 6 Passive | 7.42 | 0.49 | 6.45 | 8.39 |

**Table S2a. Mean interdialytic weight gain (IDWG; pounds) by study phase per for 2-day interdialytic intervals, per participant.**

| ID | Mean weight gain in 3 months pre-app | # of values available 3 months pre-app | Mean weight gain during app use | # of values available during app use | Mean weight gain in first 3 months passive phase | # of values available first 3 months passive |
|---|---|---|---|---|---|---|
| 1 | 5.7 | 21 | 5.4 | 18 | 6.4 | 29 |
| | 6 | 23 | 6.4 | 16 | 6.1 | 24 |
| 3 | 4.7 | 25 | 3.8 | 16 | 3.1 | 26 |
| 4 | 6.4 | 24 | 3.3 | 16 | 4.4 | 24 |
| 5 | 6.2 | 25 | 4.8 | 16 | 1.8 | 21 |
| 6 | 5.2 | 27 | 6.1 | 15 | 5.9 | 26 |
| 7 | 4.4 | 18 | 4.6 | 15 | 3.7 | 17 |
| 8 | 6.8 | 22 | 6 | 16 | 5.4 | 24 |
| 9 | 5.6 | 17 | 5.3 | 14 | 4.5 | 26 |
| 10 | 5.2 | 20 | 5.4 | 15 | 5.6 | 23 |
| 11 | 9.2 | 21 | 10.2 | 13 | 9.2 | 25 |
| 12 | 7.3 | 11 | 6.1 | 8 | 5.8 | 14 |
| 14 | 10 | 22 | 10.7 | 14 | 10.4 | 27 |
| 15 | 6.5 | 23 | 6.6 | 16 | 5.9 | 23 |
| 16 | 8 | 23 | 8 | 16 | 7.8 | 26 |
| 17 | 4.9 | 20 | 7 | 14 | 4.6 | 17 |
| 18 | 5 | 6 | 3.1 | 5 | 3.4 | 3 |
| 19 | 7.6 | 22 | 8.6 | 17 | 8.1 | 10 |
| 20 | 4.1 | 24 | 3.4 | 16 | 4.9 | 26 |
| 21 | 5 | 21 | 4.9 | 16 | 4.6 | 24 |
| 22 | 6 | 20 | 6.6 | 11 | 5.1 | 21 |
| 23 | 4.4 | 23 | 3.5 | 16 | 3.7 | 8 |

**Table S2b. Mean interdialytic weight gain (IDWG; pounds) by study phase for 3-day interdialytic intervals, per participant.**

| ID | Mean weight gain in 3 months pre-app | # of values available 3 months pre-app | Mean weight gain during app use | # of values available during app use | Mean weight gain in first 3 months passive phase | # of values available first 3 months passive |
|---|---|---|---|---|---|---|
| 1 | 8.2 | 9 | 8.5 | 4 | 8 | 1 |
| 2 | 9 | 11 | 9.9 | 8 | 8.8 | 13 |
| 3 | 7.2 | 10 | 6.8 | 8 | 6.6 | 13 |
| 4 | 9.5 | 11 | 4.9 | 8 | 6.5 | 13 |
| 5 | 7.8 | 12 | 7.3 | 8 | 3.4 | 12 |
| 6 | 7.6 | 11 | 7 | 7 | 7.4 | 13 |
| 7 | 8.2 | 9 | 9.2 | 5 | 8.1 | 7 |
| 8 | 11 | 10 | 9.5 | 8 | 8.6 | 13 |
| 9 | 6.8 | 5 | 7 | 6 | 6.6 | 13 |
| 10 | 8.9 | 13 | 8.8 | 8 | 8 | 13 |
| 11 | 11.4 | 13 | 12.3 | 9 | 12.1 | 12 |
| 12 | 8.2 | 12 | 6.7 | 6 | 6.4 | 10 |
| 13 | 10.5 | 10 | 7.3 | 5 | 11.2 | 9 |
| 14 | 13.4 | 9 | 13.1 | 8 | 12.4 | 12 |
| 15 | 9.9 | 12 | 10.5 | 7 | 10 | 14 |
| 1 | 9.8 | 12 | 10.8 | 8 | 10.8 | 13 |
| 17 | 8.4 | 9 | 7.9 | 9 | 6.6 | 13 |
| 18 | 7.9 | 7 | 7.1 | 4 | 6.3 | 8 |
| 19 | 11.4 | 12 | 10.3 | 7 | 10.1 | 12 |
| 20 | 6.1 | 13 | 6.2 | 8 | 6.5 | 13 |
| 21 | 8 | 11 | 6.7 | 8 | 6.8 | 13 |
| 22 | 9.6 | 10 | 9.7 | 8 | 9.6 | 9 |
| 23 | 7.1 | 12 | 6 | 8 | 5.9 | 5 |

**Table S3. Number of days of app use by category.**

| App use by days | n (%) |
|---|---|
| Number of days of app use | 972/1216 (79.9) |
| Number of days of app use during dialysis days | 413/515 (80.2) |
| Number of days of app use during non-dialysis days | 559/701 (79.7) |
| Number of days of app use during weekdays (M-F) | 695/870 (79.9) |
| Number of days of app use during weekends (Sa, Su) | 277/346 (80.1) |
| Number of days of app use on Mondays | 141/176 (80.1) |
| Number of days of app use on Tuesday | 140/176 (79.6) |
| Number of days of app use on Wednesday | 134/173 (77.5) |
| Number of days of app use on Thursday | 142/172 (82.6) |
| Number of days of app use on Friday | 138/173 (79.8) |
| Number of days of app use on Saturday | 135/174 (77.6) |
| Number of days of app use on Sunday | 142/172 (82.6) |

Wednesday showed the lowest usage rate, while Thursday and Saturday showed the highest. The comparison between these extremes was not statistically significant ($p=0.28$).

**Table S4. Questionnaire flow used by the study coordinator to collect participants' feedbacks on the app.**

**PART 1 – THE APPLICATION**

**Question 01**
**How frequently do you use the application?**
*Instructions: please indicate, by checking the appropriate box, how frequently you use the application*

- More than once per day
- Once per day
- At least 3 times a week
- Once a week
- Once every two weeks
- Once a month
- Less than once a month

**Question 02**
**What is your overall level of satisfaction with the application?**
*Instructions: please indicate, by circling the appropriate box, the extent to which you are satisfied or not satisfied with the application*

| Not satisfied at all | Not satisfied | Neutral | Satisfied | Very satisfied |
|---|---|---|---|---|

**Question 03**
**Below is a list of statements referring to the application.**
*Please indicate, by checking the appropriate box, the extent to which you agree or disagree with each statement.*

| | Totally disagree | Disagree | Neutral | Agree | Totally agree | Not applicable |
|---|---|---|---|---|---|---|
| The application is easy to use | ◦ | ◦ | ◦ | ◦ | ◦ | ◦ |
| I learned quickly how to use the application | ◦ | ◦ | ◦ | ◦ | ◦ | ◦ |
| The application helps me monitor accurately how much I drink | ◦ | ◦ | ◦ | ◦ | ◦ | ◦ |
| The application helps me understand why I have to reduce my fluid consumption | ◦ | ◦ | ◦ | ◦ | ◦ | ◦ |
| The application increases my motivation to meet my fluid intake target | ◦ | ◦ | ◦ | ◦ | ◦ | ◦ |
| The application helps me regulate my fluid consumption | ◦ | ◦ | ◦ | ◦ | ◦ | ◦ |
| The application helps me reach my fluid intake target | ◦ | ◦ | ◦ | ◦ | ◦ | ◦ |
| I would recommend the application to another patient | ◦ | ◦ | ◦ | ◦ | ◦ | ◦ |

## PART 2 – THE APPLICATION COMPONENTS

**Question 04**

**How often do you use the following parts of the application?**

*Instructions: for the parts of the app stated below, please indicate, by checking the appropriate box, the frequency of use.*

| | More than once per day | Once per day | At least 3 times a week | Once a week | Once every two weeks | Once a month | Less than once a month | Not applicabl e |
|---|---|---|---|---|---|---|---|---|
| Fluid intake tracker | ◦ | ◦ | ◦ | ◦ | ◦ | ◦ | ◦ | ◦ |
| Strategy planning | ◦ | ◦ | ◦ | ◦ | ◦ | ◦ | ◦ | ◦ |
| Personal statistics | ◦ | ◦ | ◦ | ◦ | ◦ | ◦ | ◦ | ◦ |
| Export of statistics | ◦ | ◦ | ◦ | ◦ | ◦ | ◦ | ◦ | ◦ |
| Health related information (tips) | ◦ | ◦ | ◦ | ◦ | ◦ | ◦ | ◦ | ◦ |
| Rewards | ◦ | ◦ | ◦ | ◦ | ◦ | ◦ | ◦ | ◦ |
| Application badges | ◦ | ◦ | ◦ | ◦ | ◦ | ◦ | ◦ | ◦ |

**Question 05**

**The following parts of the application are easy to use**

*Instructions: for the parts of the app stated below, please indicate, by checking the appropriate box, the extent to which you agree or disagree with its ease of use.*

| | Totally disagree | Disagree | Neutral | Agree | Totally agree | Not applicable |
|---|---|---|---|---|---|---|
| Fluid intake tracker | ◦ | ◦ | ◦ | ◦ | ◦ | ◦ |
| Strategy planning | ◦ | ◦ | ◦ | ◦ | ◦ | ◦ |
| Personal statistics | ◦ | ◦ | ◦ | ◦ | ◦ | ◦ |
| Export of statistics | ◦ | ◦ | ◦ | ◦ | ◦ | ◦ |
| Health related information (tips) | ◦ | ◦ | ◦ | ◦ | ◦ | ◦ |
| Rewards | | | | | | |

**Question 06**
**It is easy to learn how to use the following parts of the application**
*Instructions: for the parts of the app stated below, please indicate, by checking the appropriate box, the extent to which you agree or disagree with the ease of learning how to use it.*

| | Totally disagree | Disagree | Neutral | Agree | Totally agree | Not applicable |
|---|---|---|---|---|---|---|
| **Fluid intake tracker** | ◦ | ◦ | ◦ | ◦ | ◦ | ◦ |
| **Strategy planning** | ◦ | ◦ | ◦ | ◦ | ◦ | ◦ |
| **Personal statistics** | ◦ | ◦ | ◦ | ◦ | ◦ | ◦ |
| **Export of statistics** | ◦ | ◦ | ◦ | ◦ | ◦ | ◦ |
| **Health related information (tips)** | ◦ | ◦ | ◦ | ◦ | ◦ | ◦ |
| **Rewards** | ◦ | ◦ | ◦ | ◦ | ◦ | ◦ |

**Question 07**
**The following parts of the app are a motivation to regulate my fluid consumption**
*Instructions: for the parts of the application stated below, please indicate, by checking the appropriate box, the extent to which you agree or disagree with how it may motivate you to regulate your fluid consumption.*

| | Totally disagree | Disagree | Neutral | Agree | Totally agree | Not applicable |
|---|---|---|---|---|---|---|
| **Fluid intake tracker** | ◦ | ◦ | ◦ | ◦ | ◦ | ◦ |
| **Strategy planning** | ◦ | ◦ | ◦ | ◦ | ◦ | ◦ |
| **Personal statistics** | ◦ | ◦ | ◦ | ◦ | ◦ | ◦ |
| **Export of statistics** | ◦ | ◦ | ◦ | ◦ | ◦ | ◦ |
| **Health related information (tips)** | ◦ | ◦ | ◦ | ◦ | ◦ | ◦ |
| **Rewards** | ◦ | ◦ | ◦ | ◦ | ◦ | ◦ |

**Question 08**
**In my opinion, the following parts of the app are essential in the app:**
*Instructions: for the parts of the app stated below, please indicate, by checking the appropriate box, the extent to which you agree or disagree to the fact that it is essential to the application.*

| | Totally disagree | Disagree | Neutral | Agree | Totally agree | Not applicable |
|---|---|---|---|---|---|---|
| **Fluid intake tracker** | ◦ | ◦ | ◦ | ◦ | ◦ | ◦ |
| **Strategy planning** | ◦ | ◦ | ◦ | ◦ | ◦ | ◦ |
| **Personal statistics** | ◦ | ◦ | ◦ | ◦ | ◦ | ◦ |
| **Export of statistics** | ◦ | ◦ | ◦ | ◦ | ◦ | ◦ |
| **Health related information (tips)** | ◦ | ◦ | ◦ | ◦ | ◦ | ◦ |
| **Rewards** | ◦ | ◦ | ◦ | ◦ | ◦ | ◦ |
